# Xenotransplantation: The Way beyond and Ahead toward Clinical Application

**DOI:** 10.1155/2018/6191359

**Published:** 2018-04-04

**Authors:** Laura Iop, Vered Padler-Karavani, Emanuele Cozzi

**Affiliations:** ^1^Cardiovascular Regenerative Medicine Group, Department of Cardiac, Thoracic and Vascular Surgery, University of Padua, Padua, Italy; ^2^Venetian Institute of Molecular Medicine, Padua, Italy; ^3^Department of Cell Research & Immunology, The George S. Wise Faculty of Life Sciences, Tel Aviv University, 69978 Tel Aviv, Israel; ^4^Transplant Immunology Unit, Department of Cardiac, Thoracic and Vascular Sciences, Padua University Hospital, Padua, Italy

Instigated at the beginning of the 20th century, modern transplantation has triggered an unprecedented era for medical therapeutics. Indeed, the feasibility to successfully transplant cells, tissues, or organs derived from an allogeneic donor has opened the way to novel approaches to rescue and prolong the life of patients with end-stage organ failure. However nowadays, due to an increasing gap between the demand for organs and the limited number of human donors, allogeneic transplantation is, unfortunately, a therapeutic option that is not always available to those in need.

In the dramatic scenario of human donor shortage and increasing transplantation waiting lists, transplanting organs from other species such as the pig into man, a condition known as xenotransplantation, might represent an appealing therapeutic solution for a larger population of patients affected by organ failure. Despite the well-established similarities with regards to some aspects of anatomy and physiology between pigs and humans, transplanting pig tissues and organs into man is known to elicit an aggressive immune reaction that commonly results in premature failure of the graft.

Breakthroughs in the understanding of the mechanisms underlying xenograft rejection, that include advancements in glycoimmunology, proteomics, and molecular signaling, have enabled the development of novel modalities to prevent or delay the development of antixenograft immune responses. In fact, innovative strategies have been devised to modulate the recipient's immune response to xenografts, to reduce the immunogenic power of transplanted cells, tissues, and organs, or to create a physical barrier between the human blood and the xenograft. However, the route to the clinical application of xenotransplantation still presents many hurdles that will need to be overcome before reaching its ambitious therapeutic goal.

In this special issue, we aim to offer an overview of the state of the art of xenotransplantation with reference to the continuous innovation and potential issues in this challenging medical field. In particular, G. P. Yung and colleagues review the role of human natural killer (NK) cells in the pathophysiological mechanisms leading to xenograft rejection (in *The Role of NK Cells in Pig-to-Human Xenotransplantation*). In this context, the authors accurately describe specific molecular strategies to overcome the human NK response directed against porcine endothelial cells. As previously evidenced for NK immune responses, costimulatory molecules represent a key target of intervention to optimize immunomodulation in xenotransplantation. In the review by K. P. Samy et al. (*in The Role of Costimulation Blockade in Solid Organ and Islet Xenotransplantation*), the central role of such approaches is illustrated in enabling xenotransplantation of solid organs, as kidney, heart, and liver, as well as of islets. Specifically, advancements in terms of survival achieved in preclinical models with the introduction of monoclonal antibodies against CD154, CD40, and CTLA-4 are discussed. E. Montanari et al. cover another strategy that might possibly improve xenograft durability. This approach is represented by the protection offered to hepatocytes by cotransplantation with multipotent mesenchymal stem cells (MSCs) (in *Beneficial Effects of Human Mesenchymal Stromal Cells on Porcine Hepatocyte Viability and Albumin Secretion*). In fact, alginate-encapsulated hepatocytes demonstrated higher viability and albumin secretion upon coculture with MSCs thanks to the paracrine effect of their secreted cytokines CCL2, CXCL12, and macrophage migration inhibitory factor (MIF). Such original results are of great interest for the success of cell transplantation in the setting of acute liver failure and once again, confirm the powerful immunomodulatory and protective abilities of MSCs when applied as ancillary therapies or alone.

Besides immune response, other pitfalls in the clinical application of xenotransplantation could be represented by inappropriate organ function and growth adaptation, also developed in the long term. J. A. Shah and colleagues analyze the possible causes of post-transplant proteinuria in preclinical renal xenotransplantation and summarize the preventive strategies that are currently being tested/applied (in *Potential Pathways behind Proteinuria as well as Factors Related to Growth Discrepancies following Pig-to-Kidney Xenotransplantation*). In addition, they highlight the importance of organ size match since discrepancies may ultimately be a cause of xenograft failure. Lastly, in their review, C. G. A. McGregor and G. W. Byrne examine the current barriers hindering access of heart xenotransplantation in the clinical arena (in *Porcine to Human Heart Transplantation: Is Clinical Application Now Appropriate?*). Following an accurate description of the technological progress that has enabled the current preclinical results in nonhuman models, the remaining key preclinical studies indispensable to ensure the efficacy and safety of clinical cardiac xenotransplantation are discussed.

In this continuous cross talk between fundamental and applied research, progress is very encouraging and clinical translation of novel therapeutic solutions based on the use of animal-derived cells, tissue, or organs appears increasingly closer.

*Laura Iop* *Vered Padler-Karavani* *Emanuele Cozzi*

